# Prevalence of Chronic Pulmonary Aspergillosis in Two (2) Tuberculosis Treatment Clinics in Lagos, Nigeria: A Prospective Longitudinal Study

**DOI:** 10.1093/ofid/ofae090

**Published:** 2024-02-15

**Authors:** Adeyinka A Davies, Abiola O Adekoya, Oluwaseyi J Balogun, Iriagbonse I Osaigbovo, Augustina Nwosu, Titilola Gbaja-biamila, Olubunmi Osinupebi, Jean-Pierre Gangneux, Rita O Oladele

**Affiliations:** Department of Medical Microbiology and Parasitology, Olabisi Onabanjo University Teaching Hospital, Sagamu, Nigeria; Medical Mycology Society of Nigeria, Lagos, Nigeria; Department of Radiology, Olabisi Onabanjo University Teaching Hospital, Sagamu, Nigeria; Department of Biomedical Engineering, University of Lagos, Lagos, Nigeria; Medical Mycology Society of Nigeria, Lagos, Nigeria; Department of Medical Microbiology, School of Medicine, University of Benin, Benin City, Nigeria; Medical Mycology Society of Nigeria, Lagos, Nigeria; Central Research Laboratory, College of Medicine University of Lagos, Lagos, Nigeria; Clinical Sciences Division, Nigeria Institute of Medical Research, Yaba, Lagos, Nigeria; College of Public Health and Social Justice, Saint Louis University College of Public Health and Social Justice, Missouri, USA; Department of Medical Microbiology and Parasitology, Olabisi Onabanjo University Teaching Hospital, Sagamu, Nigeria; Laboratoire de Parasitologie et Mycologie, European Excellence Center in Medical Mycology, French National Reference Center for Chronic Aspergillosis, Centre Hospitalier Universitaire de Rennes, Rennes, France; Université de Rennes, Centre Hospitalier Universitaire de Rennes, Inserm, EHESP, IRSET (Institut de Recherché en Santé, Environnement et Travail) (UMR_S 1085), Rennes, France; Medical Mycology Society of Nigeria, Lagos, Nigeria; Department of Medical Microbiology and Parasitology, Lagos University Teaching Hospital, Idi-Araba, Lagos, Nigeria

**Keywords:** aspergillus IgG, chronic pulmonary aspergillosis, lung disease, Nigeria, pulmonary tuberculosis

## Abstract

**Background:**

Chronic pulmonary aspergillosis (CPA) is an underrecognized but common complication of pulmonary tuberculosis. In Nigeria, a tuberculosis-endemic country, there is currently no provision to monitor the development of CPA in patients treated for tuberculosis. This study determined the prevalence and incidence of CPA in Lagos, Nigeria.

**Methods:**

A prospective longitudinal study of patients with previously managed tuberculosis was conducted between June 2021 and May 2022. The study cohorts were assessed at 3-month intervals, and the following were collected: sociodemographic data, chest radiographic findings, sputum samples for fungal culture, and venous blood samples for *Aspergillus* immunoglobulin G estimation. CPA cases were determined using the case definition for resource-constrained countries. Descriptive and inferential statistics were used, and significance was set at a probability of 5% (*P* < .05).

**Results:**

Of the 141 patients recruited, 79 (56.0%) were in the retreatment and 62 (44.0%) in the posttreatment tuberculosis group. The median age (interquartile range) was 40 (30–52) years, with a male-to-female ratio of 1.1:1. Ninety-seven patients (69%) had a GeneXpert test done, of whom 63 (64.9%) were GeneXpert negative. Cough was the most common symptom, with 15 (11%) patients having hemoptysis. The rate of CPA increased steadily as the study progressed: 44 (31.2%) at commencement, 45 (34.9%) at 3 months, 49 (42.6%) at 6 months, and 51 (54.3%) at 9 months. Thus, the overall prevalence of CPA was 49.7%, and the incidence was 6.1%.

**Conclusions:**

CPA is common in Nigeria and its true burden may still be underestimated. Increased awareness of CPA as a posttuberculosis lung disease is advocated. Evaluation for CPA should be incorporated in patients’ work-up for tuberculosis.

Chronic pulmonary aspergillosis (CPA) is a disease spectrum encompassing all chronic severe *Aspergillus* infections of the lung. CPA develops in persons with underlying structural lung defects, arising from chronic obstructive pulmonary disease, sarcoidosis, nontuberculous mycobacteria infection [1], or preexisting tuberculosis. Worldwide, 3 million people are estimated to have CPA annually, with 1.2 million of these developing the condition as a sequel to tuberculosis [2], making tuberculosis the most common risk for the development of CPA. More than two-thirds of patients treated for tuberculosis will have structural changes [3], which serve as a nidus for *Aspergillus* colonization [4]. Cavity formation after tuberculosis treatment is a significant risk factor for CPA, which develops gradually over months to years, progressing at variable rates among individuals. The prevalence of cavities after tuberculosis varies, from 8% (Vietnam) to 13.7% (Iran) to 35% (Taiwan), with rates of 21%–23% in South Africa and the United States and 30% in Brazil [5].

New and relapsed tuberculosis infections were diagnosed in an estimated total of 6.4 million people in 2021 [6]. Only 63% of these were bacteriologically confirmed by smear microscopy, GeneXpert TB (Cepheid) or culture while the remaining were diagnosed clinically [6]. The signs, symptoms, and radiological features of CPA mimic those of tuberculosis, so misdiagnosis or delayed diagnosis is common, and some clinically diagnosed cases of tuberculosis may actually be CPA. However, the contribution of misdiagnosed CPA to tuberculosis prevalence estimates is not known [5]. Diagnostic and, ultimately, therapeutic delays in CPA management are made worse by the fact that awareness of the disease is low. This leads to an underestimation of the magnitude of the problem and an increase in CPA-associated morbidity and mortality rates.

Nigeria is among the 30 countries in the world with a high tuberculosis burden, accounting for two-thirds of total global cases [6]. In 2021, 590 000 new cases of tuberculosis were reported, an increase from the 452 000 reported in 2020 [6]. Possessing the highest tuberculosis burden in Africa and the sixth-highest number of tuberculosis cases globally, it is expected that the burden of CPA in Nigeria will be high. Unfortunately, treatment programs do not currently provide monitoring for CPA development in patients treated for tuberculosis. Cases of CPA are thus detected sporadically. Epidemiological studies are also rare. A cross-sectional study conducted in 2017 revealed a prevalence of 8.7% among smear/GeneXpert-negative patients receiving tuberculosis retreatment and/or those with tuberculosis treatment failure [7]. However, owing to the study design, it did not provide any information on the number of new cases per year. The present prospective study aims to determine the prevalence and at what stage CPA would develop in patients receiving retreatment for tuberculosis (retreatment group) or those who had completed tuberculosis treatment (posttreatment group).

## METHODS

### Study Design

This was a prospective longitudinal study conducted at the tuberculosis clinics in Lagos University Teaching Hospital (LUTH) and the Nigeria Institute of Medical Research, both in Lagos, Nigeria, between June 2021 and May 2022. The study population consisted of consenting adults who had previously been treated for tuberculosis 1–4 years earlier. They were clinically classified in the retreatment (relapse/reinfection) group if they were undergoing another course of antituberculosis treatment or in the posttreatment group if they had completed tuberculosis treatment. Patients in the retreatment group were recruited directly from the tuberculosis clinics, while those in the posttreatment group were recalled for participation in the study.

Written informed consent was obtained from all participants. The design of the work was approved by the institutional ethics committees of LUTH and Nigeria Institute of Medical Research (nos. ADM/DCST/HREC/APP/3868 and IRB/20/096, respectively).

### Data Collection

Basic sociodemographic data, clinical parameters, medical history, drug history, and human immunodeficiency virus (HIV) serological status were obtained from the enrolled participants.

### Laboratory Processing

At each 3-monthly visit for each patient, 5-mL venous blood samples were collected into plain nonanticoagulant bottles. The blood was allowed to clot, and the serum was separated into sterile cryotubes and stored at −80°C for *Aspergillus fumigatus*–specific immunoglobulin G (IgG) determination, using the Bordier enzyme immunoassay test kit with a cutoff of >0.8 (Bordier Affinity Products).

Expectorated sputum was collected at baseline into a sterile bottle from patients with productive cough. High-volume sputum culture was performed on Sabouraud dextrose agar containing 0.5% chloramphenicol at the mycology laboratory of the Department of Medical Microbiology and Parasitology, LUTH. Identification of *Aspergillus* species was performed phenotypically by means of macroscopic and microscopic examination.

### Case Definitions

CPA was diagnosed using the criteria for resource-constrained countries, which requires the following combinations: (1) >1 symptom that has persisted for ≥ 3 months, such as hemoptysis, persistent cough, weight loss, and/or breathlessness; (2) radiological abnormalities showing progressive cavitation, pericavitary infiltrates, and/or pleural thickening and/or fungal ball; (3) microbiological evidence of a positive *Aspergillus* IgG antibody testing and/or *Aspergillus* hyphae or *Aspergillus* growth on sputum culture; and (4) exclusion of other alternative diagnoses [4]. Pulmonary tuberculosis was diagnosed in a patient with a positive smear microscopy, culture or GeneXpert for *Mycobacterium tuberculosis* from a biological specimen [8].

Tuberculosis treatment failure was defined in a patient who is sputum smear/culture positive ≥5 months after the start of treatment or who reverted to smear positivity after an initial negative smear following treatment [9]. Relapse tuberculosis was defined as a “recurrent episode of tuberculosis (either a true relapse or reinfection) in a patient that has been previously treated and declared cured or treatment completed at the end of their most recent course of treatment” [9].

### Data Analysis

IBM Statistical Package for Social Science (SPSS) software version 25 was used for the statistical analysis. Differences were considered statistically significant at *P* < .05. Data were presented as frequency and percentages for binary and categorical variables, and median values were calculated for nonnormally distributed continuous variables. Mann-Whitney *U* tests were used for nonparametric variables and Fisher exact tests for categorical variables for CPA and non-CPA. Distributions of CPA were compared according to age and sex, using χ^2^ tests for categorical and Wilcoxon rank sum test for continuous variables; for the significance of associations, 95% confidence intervals were used, and significance was set at a probability of 5% (*P* < .05).

## RESULTS

Of 141 patients with previously managed tuberculosis, 79 (56.0%) were in the retreatment and 62 (44.0%) in the posttreatment group, and the risk of developing CPA was maximum at 6.4 per 100 tuberculosis patients per year. The median age of participants was 40 years, with a male-to-female ratio of 1.1:1. Twelve patients (7.8%) were unemployed owing to severe illness, and 40 (28.0%) admitted to smoking tobacco.

Ninety (64.0%) had undergone tuberculosis treatment twice, and only 97 (69%) had undergone a GeneXpert test. Sixty-three patients (45.0%) were GeneXpert negative, and 34 (24.0%) were GeneXpert positive. Cough was the predominant symptom in 83 patients (59.0%), 15 patients (11.0%) had hemoptysis, of whom 10 were in the retreatment group. Other clinical symptoms were fever 42 (30.0%), night sweats 38 (27.0%), chest pain 48 (34.0%), and fatigue 32 (23.0%) (Table 1). Chest radiography showed that 51 of 141 (36.0%) had cavities, 33 of 141 (23.0%) had upper lobe consolidation, 21 of 141 (15.0%) had nodules, and 25 of 141 (18.0%) had pleural thickening (Table 2).

**Table 1.** Sociodemographic and Clinical Characteristics of Study Participants

Characteristic	Participants, No. (%)^a^			*P* Value^b^
	Total (n = 141)	Non-CPA (n = 90)	CPA (n = 51)	
Tuberculosis treatment category				
Retreatment	79 (56.0)	44 (49.0)	35 (69.0)	.02
Posttreatment	62 (44.0)	46 (51.0)	16 (31.0)	
Age, median (IQR), y	40 (30–52)	42 (30–52)	39 (29–51)	.69
Sex				
Female	66 (47.0)	45 (50.0)	21 (41.0)	.31
Male	75 (53.0)	45 (50.0)	30 (59.0)	
Employment status				
Employed	89 (63.0)	59 (66.0)	30 (59.0)	.26
Retired	9 (6.4)	7 (7.8)	2 (3.9)	
Self-employed	20 (14.0)	14 (16.0)	6 (12.0)	
Student	11 (7.8)	5 (5.6)	6 (12.0)	
Unemployed	12 (8.5)	5 (5.6)	7 (13.7)	
Handling of organic or farm crops	15 (11.0)	5 (5.6)	10 (20.0)	.009
Mold growing inside house	3 (2.1)	2 (2.2)	1 (2.0)	>.99
Current smoker status	40 (28.0)	21 (23.0)	19 (37.0)	.08
No. of previous tuberculosis treatments				
1	32 (23.0)	31 (34)	1 (2.0)	<.001
2	90 (64.0)	51 (57.0)	39 (76.0)	
3	19 (13.0)	8 (8.9)	11 (22.0)	
GeneXpert results				
Negative	63 (45.0)	34 (36.0)	31 (61.0)	.008
Positive	34 (24.0)	24 (26.0)	11 (22.0)	
Not done	44 (31.0)	35 (39.0)	9 (18.0)	
Anti-*Aspergillus* IgG	…	0.38 (0.27–0.58)	2.12 (1.33–3.03)	<.001
Positive sputum culture	39 (28.0)	15 (17.0)	24 (47.0)	<.001
Symptoms				
Productive cough	83 (59.0)	49 (54.0)	34 (67.0)	.16
Hemoptysis	15 (11.0)	5 (5.6)	10 (20.0)	.009
Fever	42 (30.0)	25 (28.0)	17 (33.0)	.49
Night sweat	38 (27.0)	27 (30.0)	11 (22.0)	.28
Chest pain	48 (34.0)	29 (32.0)	19 (37.0)	.54
Fatigue	32 (23.0)	17 (19.0)	15 (29.0)	.15
MRC dyspnea scale score				
1 (Breathlessness with strenuous exercise)	69 (49.0)	54 (60.0)	15 (29.0)	<.001
2 (Shortness of Breathe when hurrying on level ground or walking up a slight hill)	55 (39.0)	24 (27.0)	31 (61.0)	
3 (Walk slower than people of my age because of breathlessness or stop for breath while walking at my own pace on a level ground?)	10 (7.1)	7 (7.8)	3 (5.9)	
4 (Stop for breathe after walking 100 yards or few minutes on level ground?)	6 (4.3)	5 (5.6)	1 (2.0)	
5 (Too breathless to leave the house or breathless after dressing or undressing?)	1 (0.7)	0 (0)	1 (2.0)	

Abbreviations: CPA, chronic pulmonary aspergillosis; IgG, immunoglobulin G; IQR, interquartile range; MRC, Medical Research Council.

^a^Data represent no. (%) of participants unless otherwise specified.

^b^ *P* values determined with Pearson χ^2^, Wilcoxon rank sum, or Fisher exact test.

**Table 2.** Sputum Culture and Radiological Findings in Study Participants

Finding	Participants, No. (%)			*P* Value^a^
	Total (n = 141)	Non-CPA (n = 90)	CPA (n = 51)	
Cavity	51 (36.0)	15 (17.0)	36 (71.0)	<.001
Fibrosis	11 (7.6)	3 (3.3)	8 (16.0)	.02
Fungal ball	10 (7.1)	1 (1.1)	9 (18.0)	<.001
Pleural thickening	25 (18.0)	5 (5.6)	20 (39.0)	<.001
Consolidation	33 (23.0)	15 (17.0)	18 (35.0)	.01
Opacity	40 (28)	9 (10.0)	31 (61.0)	<.001
Nodule				
No	120 (85.0)	83 (92.0)	37 (73.0)	.002
Yes	21 (15.0)	7 (7.8)	14 (27.0)	
Pleural effusion	8 (5.7)	4 (4.4)	4 (7.8)	.46
Lobar pneumonia	17 (12.0)	9 (10.0)	8 (16.0)	.32
Bronchiectasis	4 (2.8)	1 (1.1)	3 (5.9)	.14
Infiltration	13 (9.2)	5 (5.6)	8 (16.0)	.07
Volume loss	6 (4.3)	3 (3.3)	3 (5.9)	.67

Abbreviation: CPA, chronic pulmonary aspergillosis.

^a^ *P* values determined with Pearson χ^2^ or Fisher exact test.

### Incidence Rate, Incident Risk, and Prevalence

**Figure 1.**
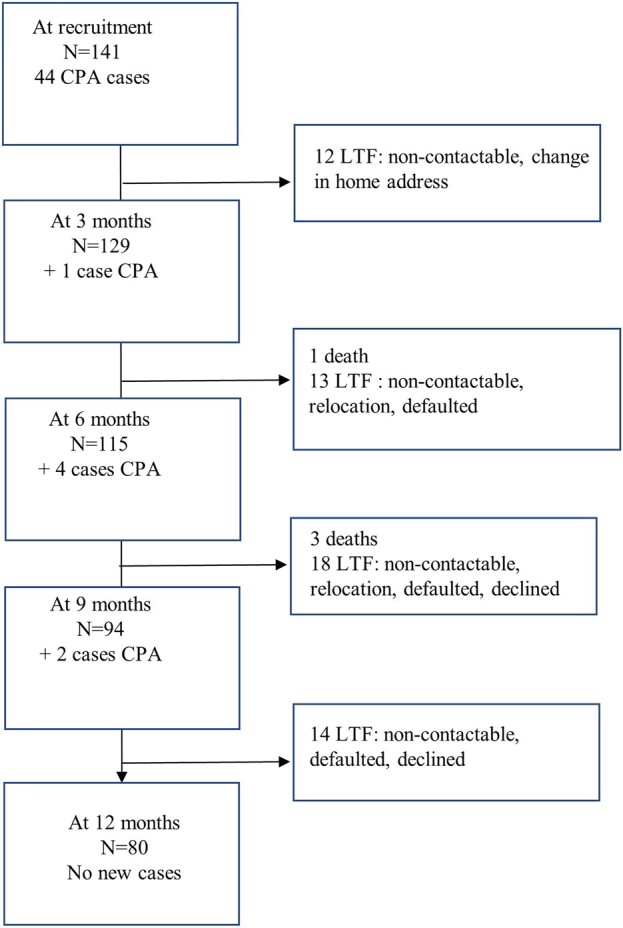
Overview of the enrolled participants. Abbreviations: CPA, chronic pulmonary aspergillosis; LTF, lost to follow-up; N, number.

An overview of patient enrollment is shown in Figure 1. At the start of the study, 44 patients met the diagnostic criteria for CPA, giving point prevalences of 24.19% and 36.71%, respectively, for the posttreatment and retreatment groups. At 12 months, there were 7 new cases of CPA. Thus, 1 year after treatment, the incidence rate for CPA in this study was specifically 6.1%, and the incident proportions for the posttreatment and retreatment groups, respectively, were 1.61% and 8.89% annually; this is approximately 2 cases of CPA per 100 per year for posttreatment and 9 cases per 100 per year for retreatment tuberculosis. The overall prevalence was 49.7%, using the number of people with CPA during the period divided by the average population at risk. However, it is pertinent to note that at consecutive clinic visits, at 3, 6, 9, and 12 months, some patients had died (4 deaths), and some did not turn up for clinic visits thus, meeting the definition for “lost to follow-up” (Figure 1).

Forty-one of the CPA participants were referred to the pulmonologis,t who placed them on itraconazole, 200 mg twice daily, for at least 6 months. Of these participants, none were on antituberculosis drugs because they had completed their treatment by the time the samples were analyzed. However, we could not contact some participants, and some CPA had already died.

### *Aspergillus* IgG Levels and Their Trends

**Figure 2.**
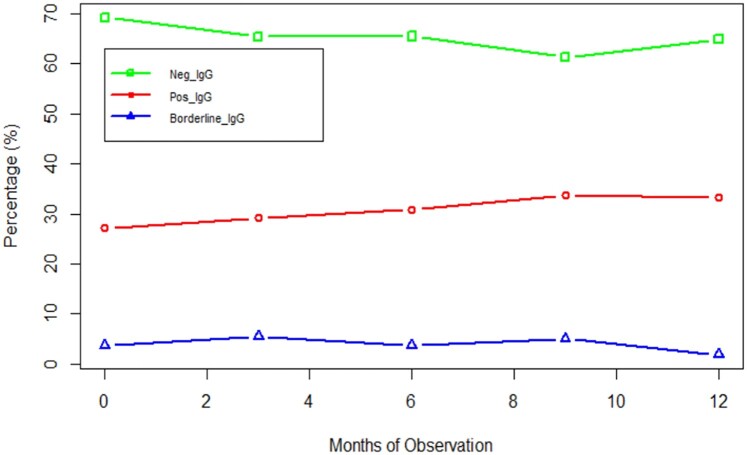
*Aspergillus*-specific immunoglobulin G (IgG) trends during the months of observation.

The trends in *Aspergillus* IgG positivity differed during the study's recruitment period. Of study participants, 31.2% (44 of 141) were positive at the start of the study, 30.2% (39 of 129) at 3 months, 31.3% (36 of 115) at 6 months, and 29.8% (28 of 94) at 9 months; there were no new positive *Aspergillus* IgG results at 12 months (Figure 2).

### High-Volume Sputum Culture Results

Fifty-nine *Aspergillus species* were cultured from the sputum of 39 participants (28%), with *Aspergillus flavus* alone isolated from 10 of 39 (25.6%) followed by *Aspergillus niger* alone in 9 of 39 (23.1%), and *A fumigatus* alone in 2 of 39 (5.1%). Mixed *Aspergillus* species were obtained in some cultures, including *A flavus* and *A fumigatus* in 10 (25.6%) *A flavus* and *A niger* in 3 (7.7%), *A fumigatus, A niger,* and *A flavus* in 2 (5.1%), and *A fumigatus* and *A niger* in 3 (7.7%). Of the 39 patients with positive *Aspergillus species* in culture, 24 (47.0%) were *Aspergillus-*specific IgG positive and 15 were *Aspergillus-*specific IgG negative (Table 3).

**Table 3.** Distribution of *Aspergillus Species* in Culture by Sex

Phenotypically Identified *Aspergillus* Species	Participants, No. (%)			*P* Value
	Total (n = 141)	Female (n = 66)	Male (n = 75)	
*A flavus*	10 (25.6)	4 (21.1)	6 (30.0)	.75
*A fumigatus*	2 (5.1)	1 (5.3)	1 (5.0)	>.99
*A niger*	9 (23.1)	4 (21.1)	5 (25.0)	>.99
*A flavus* and *A fumigatus*	10 (25.6)	5 (26.3)	5 (25.0)	>.99
*A flavus* and *A niger*	3 (7.7)	3 (15.8)	0 (0.0)	.13
*A fumigatus* and *A niger*	2 (5.1)	1 (5.3)	1 (5.0)	>.99
*A flavus, A fumigatus,* and *A niger*	3 (7.7)	1 (5.3)	2 (10.0)	>.99
Any (all positive cultures)	39 (27.7)	19 (28.8)	20 (26.7)	>.99
None	96 (68.0)	45 (68.2)	51 (68.0)	.39

### Multivariate Analysis

On multivariate analysis, tuberculosis retreatment, hemoptysis, and handling of organic or farm crops were significant predictors of CPA (Table 4).

**Table 4.** Risk Factors for Chronic Pulmonary Aspergillosis by Multivariate Analysis

Risk Factor	β Value	OR (95% CI)	*P* Value
Retreatment	1.0118	2.75 (1.19–6.34)	.02^a^
Handling of organic or farm crops	1.5757	4.83 (1.44–16.21)	.01^a^
Smoking	0.4555	1.58 (.67–3.71)	.30
Previous tuberculosis treatment	−0.7475	0.4735 (.19–1.18)	.11
Hemoptysis	1.6023	4.96 (1.49–16.51)	.009^a^
MRC dyspnea scale score	−0.6405	0.53 (.15–1.87)	.32

Abbreviations: CI, confidence interval; MRC, Medical Research Council; OR, odds ratio.

^a^Significant at *P* < .05.

## DISCUSSION

Lack of awareness among clinical and public health personnel, inadequate access to diagnostics, and lack of posttreatment care programs for follow-up after tuberculosis treatment has led to the underrecognition of CPA as a common complication of tuberculosis in tuberculosis-endemic regions. This prospective study among patients who had either completed tuberculosis treatment or were being retreated for tuberculosis revealed a point prevalence of 31.2% at baseline and a period prevalence of 63.8% after 12 months of follow-up. The study also showed that 6.1% of patients with residual lung changes after tuberculosis treatment will develop CPA annually.

The incidence of CPA in this study is similar to that reported in Uganda (6.5%), Indonesia (7.9%–13.3%), and South Korea (3.0%–7.5%) and lower than that in Ghana (10.5%) [1, 10–12]. The earliest study documenting CPA from the United Kingdom reported an incidence of 22% in those with cavities after antituberculosis therapy [13]. Residual lung cavities and other structural lung defects are documented risks for CPA, with lung cavities, reported as the most common abnormalities [3, 10].

Both point and period prevalences of CPA recorded in this study were much higher than the 8.7% reported from a cross-sectional study conducted within the same clinics (plus an additional site in southwest Nigeria), some 6 years earlier [7]. The differences in prevalence may be attributed to the differences in study design (prospective vs cross-sectional), variability in the study population, and serological kits used for the analysis (Bordier vs ImmunoCAP [Thermo Fisher Scientific]). In 2020, the World Health Organization estimated that 452 000 Nigerians had tuberculosis, of whom 135 000 had relapse/retreatment tuberculosis [6]. The findings of the index study stoke curiosity as to how many of these relapse/retreatment cases and even the new cases may actually be CPA or tuberculosis-*Aspergillus* coinfections and imply that the true extent of CPA in Nigeria may be underestimated.

In terms of sociodemographic parameters, there was a higher proportion of male than female participants among those with CPA. This is not surprising, since other studies on CPA have also confirmed male predominance [10, 12, 14]. Men may be more susceptible to the disease owing to combinations of biological and behavioral factors, such as delay in seeking medical care, consumption of alcohol, and tobacco smoking [15]. In addition, the proportion of patients with CPA who handled organic matter or farm crops was higher than that in patients without CPA, which may suggest greater exposure to *Aspergillus* spores, and this difference was statistically significant. None of these associations (male sex, smoking, etc) were statistically significant. However, they are worthy of mention and may require a larger sample size to demonstrate significance. The sociodemographic profile of patients with CPA in this study fits the paradigm of CPA as a disease that affects mostly young men in their prime, leading to loss in productivity with a possible negative impact on the national economy.

In this study, patients with CPA diagnosed complained of productive cough, hemoptysis, chest pain, night sweats, and fatigue, all symptoms commonly seen in tuberculosis. Because CPA and tuberculosis have similar clinical presentations, distinguishing between them is difficult, and patients with persistent respiratory symptoms after tuberculosis therapy should be evaluated for CPA [16]. Although cough was the predominant symptom in this study, it was not significantly associated with CPA. On the other hand, hemoptysis was the least common symptom, but its association with CPA was significant. This is similar to findings from Ghana and Brazil [12, 14].

Persistent hemoptysis and cough lasting ≥3 months during or after tuberculosis treatment might suggest CPA [2, 12], but, radiological and microbiological confirmation are necessary; a negative GeneXpert result, an abnormal chest radiograph, and a positive *Aspergillus* IgG serological result and/or *Aspergillus* in sputum culture help confirm a diagnosis of CPA. In addition, Denning et al [4] showed that some patients with CPA may be asymptomatic, and only chest imaging will show abnormalities or progression of the disease, as was observed in some of the asymptomatic posttreatment participants in the index study.

CPA and tuberculosis also share some similarities in terms of radiological appearance. Pleural thickening, para cavitary fibrosis, or the increase in the size of the cavities or the formation of a new cavity strongly indicate CPA [1]. Fibrosis, lung infiltrates, and bronchiectasis may appear in both tuberculosis and CPA, while pleural effusion is more indicative of tuberculosis [2].

Although rare, CPA and *M tuberculosis* coinfection does occur [10]. The current study found 7 participants (16.3%) with CPA and *M tuberculosis* coinfection. The prevalence of coinfection was 26.7% *M tuberculosis*–positive Ghanaians, 20% in HIV-positive Ghanaians [12], 6.5% in HIV-positive Nigerians [7], and 2.9% in Vietnam and Pakistan [17, 18]. *M tuberculosis* and *Aspergillus* species are opportunistic pathogens of public health importance which progressively affect the lung parenchyma. In tuberculosis-endemic countries, it is pertinent that all patients with tuberculosis must concurrently undergo screening for CPA [17–19].

Commercially available *Aspergillus* antibody kits, including the Bordier assay used in this study, are targeted against *A fumigatus,* since it has been documented as the predominant species causing human aspergillosis [7, 10, 12]. After the baseline, new cases of CPA were diagnosed with serial *Aspergillus* serology and radiological abnormalities specific for CPA (based on the diagnostic criteria). Sputum culture was performed once in those with productive cough. However, in this study, *Aspergillus flavus* was the predominant species isolated from high-volume sputum cultures. This was followed by *A niger,* before *A fumigatus*. Similarly, other African and Asian studies report more non*-fumigatus* species, with *A niger* accounting for 36.8% and *A flavus* for 47.2% of isolates from sputum cultures in patients with CPA from Ghana and Pakistan, respectively [12, 18]. The sensitivity of the *Aspergillus* IgG assay may be low for non*-fumigatus* infection [7]. This could explain 10 patients who eventually had CPA diagnosed despite *Aspergillus-*specific IgG levels <0.8 but had sputum cultures positive for *A flavus* (n = 8) and combined *A niger* and *A flavus* (n = 2).

These observations underscore the importance of including sputum fungal culture in the diagnostic workup for CPA, despite its low sensitivity, especially in areas where non-*fumigatus* species appear to be dominant. Furthermore, false-negative results from antibody kits may also occur in HIV-positive patients or those with subtle immunodeficiency states, because of their reduced capacity to mount sufficient antibody response against *Aspergillus* infection [7, 20]. This scenario was also illustrated in the index study, where 1 patient with HIV infection had a fungal ball at chest radiography but also had a negative *Aspergillus-*specific IgG result. Thus, the *Aspergillus-*specific IgG assays, though vital to CPA diagnosis, may lead to diagnostic delays and even misdiagnosis if relied on solely, further contributing to underestimating CPA burden. Interestingly, the Medical Research Council [21] dyspnea scale score revealed that both patients with and those without CPA had underlying functional respiratory impairments and low quality of life, thus underscoring the prognostic importance of early diagnosis and treatment to prevent disease progression.

The modality of imaging used in this study was one of the major limitations. Chest radiography, while relatively inexpensive, does not provide the best resolution, and computed tomography is better at showing lung changes. In addition, owing to limited funds, serial chest imaging, which would have shown the progression of lesions. could not be done. Despite use of the high-volume culture technique, the sensitivity of the sputum fungal cultures was low, and this also limited the ability to identify the species of *Aspergillus* responsible in all cases of CPA. Finally, a substantial number of patients were lost to follow-up, for a variety of reasons. This might mean that the burden of CPA in the cohort was underestimated. In spite of these limitations, the prospective nature of the study was its strength, since it was able to demonstrate that patients who have received treatment for tuberculosis progressively run the risk of developing CPA. Continued monitoring of these patients is thus warranted, and future studies should determine the maximum duration for follow-up.

In conclusion, CPA is common in Nigeria. Its true burden is probably still underestimated, and patients who have undergone tuberculosis treatment represent a population at very high risk for CPA. Both patients with suspected and those with confirmed tuberculosis require concurrent evaluation for CPA, based on their symptom profile and the radiological and microbiological diagnostic modalities used. In addition, confirmed cases of tuberculosis should be monitored for the evolution of CPA during and after completion of the antituberculosis regimen, especially if symptoms persist or worsen. There is a need to continuously raise awareness and increase clinicians’ level of suspicion for CPA. Furthermore, posttreatment patients require long-term follow-up that systematically incorporates screening for posttuberculosis lung diseases such as CPA, among others.
